# Correlation between maternal and neonatal blood Vitamin D level: Study from Pakistan

**DOI:** 10.1111/mcn.13028

**Published:** 2020-08-20

**Authors:** Shahida Rabbani, Saima Afaq, Sheraz Fazid, Maria Ishaq Khattak, Yasir Mehmood Yousafzai, Syed Hamid Habib, Nicky Lowe, Zia Ul‐Haq

**Affiliations:** ^1^ Institute of Public Health and Social Sciences Khyber Medical University Peshawar Paksitan; ^2^ School of Public Health, Faculty of Medicine Imperial College London London UK; ^3^ School of Sport and Nutritional Sciences University of Central Lancashire Preston UK; ^4^ Institute of Health and Well‐being University of Glasgow Glasgow UK

**Keywords:** 25 (OH) Vitamin D, 25(OH)D, deficiency, LMIC, neonatal, newborns, nutrition, Pakistan, pregnancy, pregnant mothers, South Asia

## Abstract

In Pakistan, there is limited evidence for the levels and relationship of 25 (OH) Vitamin D (25(OH)D) status in pregnant women and their newborns, while the association between maternal 25(OH)D and newborn anthropometric measurements remains unexplored. Sociodemographic data were collected from 213 pregnant mothers during their visit to a tertiary care hospital at the time of childbirth. Anthropometric measurements were performed on all mothers and their newborns and blood samples collected from both for 25(OH)D levels. Participants were classified into two groups according to their 25(OH)D status: sufficient (25(OH)D ≥50 nmol L^−1^) and deficient (25(OH)D <50 nmol L^−1^). Simple and multiple regression models were used for analysis. Among 213 pregnant women, prevalence of 25(OH)D deficiency was 61.5%, and their newborn was 99.5% (mean 25(OH)D levels: 46.3 [11.3] and 24.9 [5.4] nmol L^−1^, respectively). Maternal sociodemographic characteristics were similar between 25(OH)D deficient and sufficient mothers, whereas newborn 25(OH)D levels were significantly lower in the former (22.60 [4.53] vs. 27.67 [3.82] nmol L^−1^, respectively, *P* < 0.001). There was a strong positive association between maternal and newborn 25(OH)D levels (*r*, 0.66; *r* ^2^, 43%, *B* [SE], 0.3 [0.02]; *P* < 0.001). Association of maternal 25(OH)D levels with newborn weight, length and head circumference was not significant (all *P* > 0.05). Our study shows a high prevalence of 25(OH)D deficiency in pregnant women and their newborns and a strong positive association between maternal and newborn 25(OH)D levels. Findings of this study indicate the importance of maintaining sufficient 25(OH)D levels during pregnancy.

**Key messages**

- Vitamin D deficiency is very high in pregnant mothers and their newborns.
- Our findings support the evidence that newborns are dependent on their mothers for Vitamin D supply.
- Antenatal visits should include education on the safety and importance of Vitamin D supplementation and sufficient sunlight exposure during pregnancy.
- Pregnancy specific Vitamin D cut‐offs are required for optimal health of the mothers and the newborns and to enable comparisons.
- Prospective and interventional studies are required to investigate the optimum Vitamin D requirement for pregnant mothers and their newborns and determine maternal Vitamin D associations with newborn outcomes.

## 1 INTRODUCTION

25 (OH) Vitamin D (25(OH)D), a fat soluble secosteroid (Veena et al., 2016), contributes towards numerous physiological (Carlberg, 2014) and metabolic (Bikle, 2014) processes in human body. Role of 25(OH)D in pregnant women and the newborn has been well established (Bowyer et al., 2009). The effects of Vitamin D deficiency in pregnant women include backache/leg cramps (Mansoor et al., 2010; Mansouri, Mirghafourvand, Charandabi, & Najafi, 2017),serious metabolic diseases (Bodnar et al., 2007; Mutlu et al., 2015) and adverse newborn outcomes (Camargo et al., 2007; Javaid et al., 2006).

A deficiency of 25(OH)D is recognized as a global public health problem (Holick & Chen, 2008)^.^ and is reported in both low–middle‐income countries (Aslam, Sattar, Masood, & Qudsia, 2012; Cashman, Sheehy, & O'Neill, 2019; Roth et al., 2018) as well as the industrialized countries including the United States (Liu, Baylin, & Levy, 2018; Parva et al., 2018) and Europe (Domaracki et al., 2016; Gellert, Ströhle, Bitterlich, & Hahn, 2017). International studies have shown that 25(OH)D levels in the newborn are directly dependent on the maternal 25(OH)D level (Jacquemyn, Ajaji, & Karepouan, 2013; Nobles, Markenson, & Chasan‐Taber, 2015; Pludowski et al., 2011)^.^ Deficiency of 25(OH)D in pregnant women may, therefore, result in 25(OH)D deficiency in their newborns (Hollis & Wagner, 2017; Kiely et al., 2017)^,^ which may, subsequently, negatively affect the anthropometric parameters, including birth weight, length and head circumference, in the newborn (Gernand, Simhan, Klebanoff, & Bodnar, 2013; Jacquemyn et al., 2013; Sathish, Sajeethakumari, Padma, Balakrishnan, & Muthusami, 2016; Veena et al., 2016). The findings are, however, inconsistent (Autier, Boniol, Pizot, & Mullie, 2014)^.^

In Pakistan, there is limited available data on the levels of 25(OH)D in pregnant mothers and their newborns. The National Nutrition Survey (NNS) of Pakistan 2011 (Pakistan & Khan, 2011) was the first time when 25(OH)D deficiency was recorded on a large scale in Pakistan. Overall, prevalence of 25(OH)D deficiency (defined as 25(OH)D levels <20 ng ml^−1^) in pregnant women was 68.9% (73.9% in urban areas and 67.2%in rural areas) with considerable variation among the different provinces (Punjab 71.1%, Sindh 66.9%, Khyber Pakhtunkhwa 63.8%, Balouchistan 43.6%, AJK 73.4% and Gilgit Baltistan 76.1%).

Evidence regarding the strength of relationship between maternal and newborns 25(OH)D levels, from Pakistan, also shows marked variation (Hossain et al., 2011; Karim, Nusrat, & Aziz, 2011; Khan, Iqbal, Naureen, Dar, & Ahmed, 2012; Riaz et al., 2016), while the association between maternal 25(OH)D levels and anthropometric measurements of the newborn remains unexplored. This study aims to measure 25(OH)D status among pregnant women and their newborns and explore the relationship between maternal 25(OH)D levels and the 25(OH)D levels and anthropometric measurements (weight, length and head circumference) of the newborn.

## 2 METHODS

### 2.1 Data source

We conducted a cross‐sectional study on healthy pregnant women and their newborns, who visited the Department of Obstetrics and Gynaecology, Military Hospital Rawalpindi from 1 September 2015 to 28 February 2016. Women with preterm delivery, multiple pregnancy, pregnancy‐induced hypertension, chronic illness, diabetes mellitus, chronic heart disease, hepatic or renal impairment, tuberculosis and fat malabsorption diseases and women using medications known to affect bone metabolism, for example, anti‐convulsant, corticosteroids, rifampicin, cholestyramine, isoniazid and theophylline, were excluded from the study. Additionally, mothers having newborns with congenital anomalies were also excluded. Purposive sampling technique was used for recruitment of the study participants.

Rawalpindi is the fourth largest city of Pakistan located in the northernmost part of the Punjab province. Latitude and longitude coordinates are 33.626057 and 73.071442, respectively. The city of Rawalpindi includes both rural and urban areas and people from low‐, middle‐, and high‐income backgrounds.

Ethical approval was provided by the Ethical and Research Board of the Military Hospital Rawalpindi, and informed written consents were taken from all participants upon admission at the hospital during (prelabour).

### 2.2 Data collection

All participants completed a pro forma, at the time of admission to the hospital, including questions on demographics, monthly income, education, typical daily duration of sun exposure, type of clothing worn when outdoors, colour of the skin and use of 25(OH)D supplements.

Anthropometric measurements were performed on both the mother (at the time of admission) and the newborn (immediately after birth) using standardized scales. Maternal anthropometry included weight and height measurements. Weight was measured to the nearest 0.1 kg using digital scales mounted on a hard and flat surface. Height was measured to the nearest 0.1 cm, using a stadiometer, mounted on a hard and flat surface. Newborn anthropometry included weight, length and head circumference. Newborn weight was measured to the nearest 5 g using baby scales. Length and height were measured to the nearest 0.1 cm using a nonstretchable measuring tape.

Blood samples from the mothers and cord blood of their newborns were collected immediately after the delivery. All maternal and newborn blood samples were sent to the biochemistry laboratory of the Armed Forces Institute of Pathology (AFIP) Rawalpindi, for estimation of 25(OH)D levels, where they strictly follow internal and external quality control checks (https://www.afip.gov.pk/index.php?page=accreditation). Blood samples were collected by trained nurses and sent immediately to the lab for analysis according to the available SOPs of the hospital. Blood samples were analysed by the liquid chromatography‐tandem mass spectroscopy assays. The test principle is based on an electro‐chemiluminescence immuno assay (ECLIA). The assay uses a 25(OH)D‐binding protein (VDBP) as capture protein that binds to Vitamin D3 (25‐hydroxyvitamin).

### 2.3 Definitions

Participants were classified into two groups according to their 25(OH)D levels: sufficient, defined as 25(OH)D ≥ 50 nmol L^−1^, and deficient, defined as 25(OH)D <50 nmol L^−1^ (Ross, Taylor, Yaktine, & Del Valle, 2011)^.^ Low birth weight (LBW) was defined as a birth weight of less than 2,500 g (up to and including 2,499 g), as per the World Health Organization (WHO) (Brämer, 1988). Body mass index (BMI) (kg m^−2^) was categorized into underweight (<18.5), normal weight (18.5 to <25), overweight (25.0 to <30) and obese (30.0 or higher) using standard cut‐points (Organización Mundial de la Salud, 2000).

Monthly income was categorized into low‐, middle‐ and high‐income groups according to the World Bank's classification of income groups based on gross national income (GNI) per capita (The World Bank, 2017). Educational status was classified into two categories: illiterate, defined as having no formal schooling and unable to read and write, and literate, defined as any number of years of formal schooling and able to read and write.

Due to the sociocultural reasons, sun exposure means the exposure of hands and/or arms, feet, face and neck (bare skin) to the sunlight in this setting. Through literature search, it was identified that the minimum duration of sun exposure, for any beneficial effects, is usually 10 min. Hence, this cut‐off of 10 min day^−1^ was chosen to classify the women as adequately or inadequately sun exposed for this study.

### 2.4 Statistical analysis

Continuous data were expressed as mean (standard deviation), and categorical data as percentage (%). Distribution of the study variables was assessed using histograms. Univariate tests of the differences were carried out between 25(OH)D sufficient and deficient mothers using the independent samples *t* test and chi‐squared test for continuous and categorical variables, respectively. Pearson's correlation coefficient was used for initial univariate assessment of the relationship between the 25(OH)D status of mother and newborn. Effect of possible confounders on the relationship between maternal and newborn 25(OH)D levels was assessed using multiple linear regression. Relationship between maternal and newborn anthropometric measurements (weight, length and head circumference) was determined using multiple linear regression. *P* value of less than 0.05 was considered statistically significant. Mothers taking Vitamin D supplements were excluded from the final analysis. Statistical analyses were performed using STATA 14.

## 3 RESULTS

### 3.1 Characteristics of participants

We studied a total of 213 healthy pregnant mothers (attending the hospital at the time of delivery) and their 213 newborns (*N* = 416). Table 1 presents the characteristics of our study participants. Among these participants, ~67% were aged less than 30 years, and ~33% were aged 30 or above. Mean maternal age was 27.8 (4.1) years. The women were, on average, multiparous, and the mean gestational age was ~38 (1.7) weeks. Mean maternal body mass index was 27.8 (3.2) kg m^−2^. The proportion of mothers with a daily sun exposure of less than 10 min was 16%, whereas 84% of the mothers had daily sun exposure equal to or more than 10 min. The prevalence of 25(OH)D deficiency in the mothers was 61.5%, and mean 25(OH)D level was 46.3 (11.3) nmol L^−1^. Around 19% (*n* = 41) of the mothers had received 25(OH)D supplements and were therefore excluded from further analysis.

**TABLE 1:** Overall characteristics of the pregnant mothers and their newborns

Characteristics	Frequency *n* (%)
Maternal characteristics (*N* = 213)	
Vitamin D status (nmol L^−1^)	
Deficient (<50)	131 (61.5)
Sufficient (≥50)	82 (38.5)
Age of mother (years)	
Less than 30	143 (67.1)
Equal to or more than 30	70 (32.9)
Parity (*n*)	
Uniparous	56 (26.3)
Multiparous	157 (73.7)
Gestational age (weeks)	
Less than 36	12 (5.76)
Equal to or more than 36	201 (94.4)
BMI (kg m^−2^)	
Normal weight	28 (13.1)
Overweight	121 (56.8)
Obese	64 (30.0)
Clothing veil	
No	182(85.4)
Yes	31(14.6)
Average monthly income (Pak rupees)	
Low	63 (28)
Middle	105 (46.67)
High	57 (25.33)
Backyard/garden	
No	108 (50.7)
Yes	104 (48.8)
Colour of skin	
Fair	116 (54.5)
Dark	97 (45.5)
Vitamin D supplements	
No	172 (80.8)
Yes	41 (19.2)
Sun exposure (min/day^−1^)	
Less than 10	34 (16.0)
Equal to or greater than 10	179 (84.0)
Newborn characteristics (*N* = 213)	
Gender of the baby (%)	
Male	111 (52.1)
Female	102 (47.9)
Vitamin D status (nmol L^−1^)	
Deficient (<50)	212 (99.5)
Sufficient (≥50)	1 (0.5)
Low birthweight (<2.5 kg)	
No	189 (88.7)
Yes	24 (11.4)
Length (cm)^a^	47.4 (2.8)
Head circumference (cm)^a^	33.9 (2.2)

^a^ Results are presented as Mean (SD).

Abbreviation: BMI, body mass index.

The proportion of the male and female newborns was 52.1% and 47.9%, respectively. Mean 25(OH)D levels were 24.9 (5.4) nmol L^−1^, and 99.5% of the newborns were 25(OH)D deficient. The average birthweight was 2.9 (0.5) kg with 11.4% prevalence of low birthweight. Mean length and head circumference of the newborns were 47.4 (2.8) and 33.9 (2.2) cm, respectively (Table 1).

Maternal and newborn clinical characteristics were compared between the 25(OH)D sufficient and deficient mothers **(**Table 2 **).** Sociodemographic characteristics were similar between the two groups (all *P* > 0.05). Compared with the 25(OH)D sufficient mothers, the newborn 25(OH)D levels were significantly lower among the 25(OH)D deficient mothers (*P* < 0.001). There was no significant difference in other newborn characteristics between the two groups (Table 2).

**TABLE 2:** Characteristics of the study participants by mother Vitamin D status

*N*	Deficient (<50 nm L^−1^)	Sufficient (≥50 nm L^−1^)	*P*
	Mean (SD)/*n* (%)	Mean (SD)/*n* (%)	
	105	67	
Maternal characteristics			
Age (years)	27.62 (3.87)	28.09 (4.64)	0.5
Gestational age (weeks)	38.20 (1.58)	37.93 (1.57)	0.3
Height (meters)	1.55 (0.05)	1.55 (0.04)	0.2
Weight (kg)	67.09 (6.54)	68.18 (7.41)	0.3
BMI (kg m^−2^)	27.61 (2.98)	28.50 (3.29)	0.07
Monthly income (rupees)	23,504.76 (4,372.39)	24,074.63 (4,875.15)	0.4
Parity			0.2
Uniparous	21 (20.0)	19 (28.4)	
Multiparous	84 (80.0)	48 (71.6)	
Education			0.2
No formal education	48 (45.7)	38 (56.7)	
Educated	57 (54.3)	29 (43.3)	
Daily sun exposure (%)			0.6
Less than 10 min	17 (16.2)	9 (13.4)	
Equal to or more than 10 min	88 (83.8)	58 (86.6)	
Clothing veil			0.3
No	86 (81.9)	59 (88.1)	
Yes	19 (18.1)	8 (11.9)	
Colour of skin			0.7
Fair	63 (60.0)	38 (56.7)	
Dark	42 (40.0)	29 (43.3)	
Newborn characteristics			
Vitamin D level (nmol L^−1^)	22.60 (4.53)	27.67 (3.82)	<0.001
Length of baby (unit)	47.17 (2.73)	46.96 (2.64)	0.6
Weight of baby (unit)	2.89 (0.52)	2.86 (0.50)	0.6
HC of baby (unit)	33.76 (2.14)	33.81 (2.12)	0.9
Male %	58 (55.2)	34 (50.7)	0.6

*Note*: Mothers with Vitamin D supplementation have been excluded from analysis.

Abbreviation: BMI, body mass index.

### 3.2 Association between maternal and newborn Vitamin D status

**FIGURE 1:**
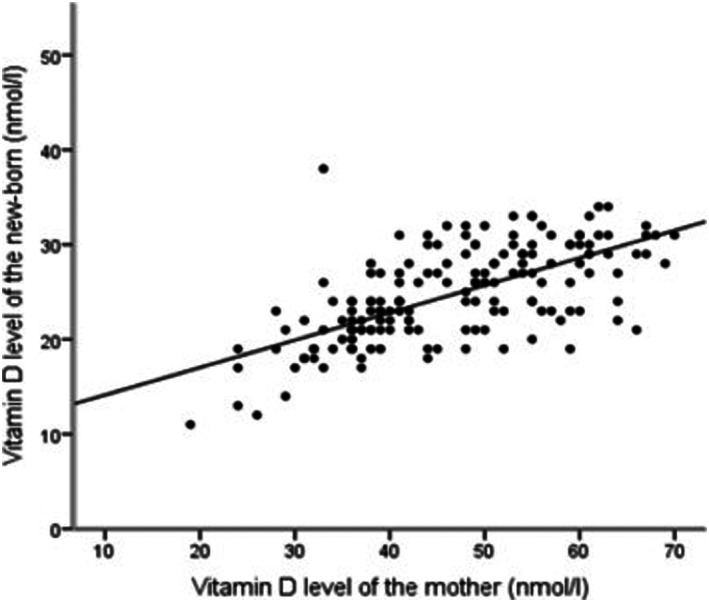
Scatter plot showing the correlation between Vitamin D status of mother (*N* = 213) and the Vitamin D status of her newborns (*N* = 213), *r* = 0.66, *r* ^2^ = 0.43, *P* < 0.0001

The univariate relationship (Figure 1) between 25(OH)D levels of mother and her newborn baby showed a strong positive correlation (*r* = 0.66, *P* < 0.001). 25(OH)D status of the mother explained 43% variation in the 25(OH)D status of the newborn (coefficient of determination: *r* ^2^ = 0.43, *P* < 0.001). We further studied the effect of maternal characteristics (age, gestational age, BMI, education, monthly income, skin colour, parity and sun exposure) on the association between maternal and newborn 25(OH)D levels using multiple linear regression (Table 3). Maternal 25(OH)D level was defined as the predictor, whereas newborn 25(OH)D level was the outcome. There was a positive association between maternal and newborn 25(OH)D levels (*B* [SE], 0.289 [0.026]; *P* < 0.001). The association between the two parameters remained significant even after adjustment for the maternal demographic and clinical characteristics (all *P* < 0.001, Table 3).

**TABLE 3:** Effect of maternal characteristics on the relationship between maternal and newborn Vitamin D levels

Model	Adjusted for	Relationship between maternal and newborn Vitamin D levels	
		*B* (SE)	*P*
1	Unadjusted	0.289 (0.025)	<0.001
2	Model 1 + maternal age	0.289 (0.026)	<0.001
3	Model 2 + parity	0.289 (0.026)	<0.001
4	Model 3 + gestational age	0.290 (0.026)	<0.001
5	Model 4 + BMI	0.303 (0.026)	<0.001
6	Model 5 + monthly income	0.299 (0.026)	<0.001
7	Model 6 + sun exposure	0.301 (0.026)	<0.001
8	Model 7 + education	0.302 (0.027)	<0.001

Results are presented as Beta (SE) from multiple linear regression (*N* = 213)

Abbreviation: BMI, body mass index.

### 3.3 Association between maternal and newborn anthropometric measurements

The relationship between maternal and newborn anthropometric measurements (weight, length and head circumference) was determined using multiple linear regression. Results were presented before and after adjustment for age, gestational age, BMI, education, monthly income, skin colour, parity and sun exposure (Table 4). Both unadjusted and adjusted analysis did not reveal a significant association between maternal 25(OH)D levels and any of the anthropometric measurements: newborn weight: *B* [SE]: −0.001 (0.003), *P* = 0.8 and 0.003 (0.003), *p* = 0.3; length: *B* [SE]: 0.018 (0.018), *P* = 0.3 and 0.019 (0.019), *P* = 0.3; and head circumference: *B* [SE]: 0.008 (0.015), *P* = 0.6 and 0.013 (0.014), *P* = 0.3, for unadjusted and adjusted analysis, respectively.

**TABLE 4:** Relationship of maternal Vitamin D levels with newborn anthropometric measurements (*N* = 213)

Newborn anthropometric parameters	Unadjusted		Adjusted	
	*B* (SE)	*P*	*B* (SE)	*P*
Weight (kg)	−0.001 (0.003)	0.8	0.003 (0.003)	0.3
Length (cm)	0.018 (0.018)	0.3	0.019 (0.019)	0.3
Head circumference (cm)	0.008 (0.015)	0.6	0.013 (0.014)	0.3

*Note*: Results are presented as Beta (SE) from multiple linear regression, before and after adjustment for possible confounders (maternal age, gestational age, BMI, education, monthly income, skin colour, parity and sun exposure).

## 4 DISCUSSION

Our study shows a high prevalence of 25(OH)D deficiency in mothers of newborn infants, women (61.4%) and their newborns (99.5%). Strong positive association was observed between maternal and newborn 25(OH)D levels (*r*, 0.66; *r* ^2^ 43%; *B* [SE], 0.3 [0.02]; all *P* < 0.001). This association between the maternal and newborn 25(OH)D levels was independent of maternal characteristics. No characteristics of the mother (age, parity, clothing veils, sunlight exposure and anthropometry) and their newborns (weight, length and head circumference) were significantly associated with the 25(OH)D deficiency.

Such high prevalence of 25(OH)D deficiency indicates the importance of Vitamin D supplementation in both the mothers and their newborns. Our study supports the positive association between maternal and newborn cord blood 25(OH)D levels, controlled for confounding variables. Blood samples from the mothers were taken in the months of September to February, which corresponds to autumn–winter season in Rawalpindi (Pakistan) where the average length of day and the sun exposure is less than compared with the summer season. It may therefore be likely that the high prevalence of 25(OH)D deficiency is associated with seasonality, as reported in previous studies too (Goswami et al., 2000; Sheikh, Saeed, Jafri, Yazdani, & Hussain, 2012). Maternal demographic and physical characteristics did not have any effect on the 25(OH)D levels of the mothers and their newborns. Other possible population‐specific genetic and environmental factors need to be identified that may have influenced the newborn 25(OH)D status.

Several studies, from other countries (Eggemoen et al., 2017; Leffelaar, Vrijkotte, & Van Eijsden, 2010; Rodriguez et al., 2015), reported no relationship between maternal 25(OH)D levels and newborn anthropometric parameters. An observational study on 2,146 pregnant women found a significant relationship between maternal 25(OH)D levels and newborn birthweight only after adjusting for several confounders, including ethnicity, pre‐pregnancy BMI, trimester at maternal blood withdraw and study site (Gernand et al., 2013). In contrast, a prospective study conducted in Spain reported no association between maternal and newborn birthweight in either adjusted or unadjusted analysis (Rodriguez et al., 2015). Furthermore, research studies observed there was no relationship between maternal 25(OH)D inadequacy with the infant's height and head circumference (Ong et al., 2016; Wierzejska et al., 2018).

Nevertheless, some authors have suggested the presence of an association between maternal 25(OH)D levels and newborn anthropometric measurements. A study from India (Kaushal & Magon, 2013) reported lower weight (by 480 g), length (by 9.5 cm) and head circumference (by 4.5 cm) in the newborns of Vitamin D deficient mothers compared with the mothers with sufficient Vitamin D levels. A study conducted in the United States reported a similar relationship, although the difference in newborn weight was smaller (by 176 g) (Nobles et al., 2015)^.^ Moreover, another study reported that newborns whose mothers received Vitamin D had greater head circumference compared with babies of mothers who did not get Vitamin D (Abbasian et al., 2016)^.^ A meta‐analysis of observational study revealed that newborns of mothers with 25(OH)D levels of <37.5 nmol L^−1^ during pregnancy had lower birth weight (random weighted mean difference [95% CI] −130.92 g [186.69 to 75.14 g])’ however, there was no significant effect on birth length and head circumference (Aghajafari et al., 2013). A systematic review suggested that evidence from the available literature is not sufficient to confirm an association between maternal 25(OH)D levels and newborn outcomes (Harvey et al., 2014). The evidence, therefore, regarding the relationship between maternal 25(OH)D and newborn anthropometric measurements. is inconclusive.

Several limitations might have biased our results. Cut‐off value of 50 nmol L^−1^ was used in this study to classify the 25(OH)D sufficient and deficient participants. Many investigators support the use of 50 nmol L^−1^ as the cut‐off, below which the parathyroid hormone starts to rise sharply (Holick, 2006; Holick & Chen, 2008), and the 25(OH)D levels are not adequate to maintain bone and overall health in healthy individuals (Ross et al., 2011). Vitamin D levels were assessed only at the time of delivery, which do not represent the levels for the entire duration of pregnancy. Diet assessment, identified as one of the determinants of 25(OH)D status (Lips, van Schoor, & de Jongh, 2014), was not performed. Weight of the mothers was assessed at the time of admission (third trimester of pregnancy), which may account for the high prevalence of obesity in our study. The current study was conducted in a hospital setting where mostly families of army employee visit, so caution is advised before generalizing the results.

## 5 CONCLUSION

We identified a very high burden of Vitamin D deficiency in pregnant mothers and their newborns, exposing them to a higher risk of health problems. Our findings are coherent with the current literature (Bhimji, Naburi, Aboud, & Manji, 2018; Mustafa, Asadi, Iqbal, & Bashir, 2018; Özdemir et al., 2018; Sheikh et al., 2012; Urrutia‐Pereira & Solé, 2015) and support the evidence that newborns are dependent on their mothers for the supply of Vitamin D during early life (Mulligan, Felton, Riek, & Bernal‐Mizrachi, 2010; Sharif, Farasat, Shoaib, Saqib, & Fazal, 2013). Hence, we suggest that Vitamin D supplementation should be considered for 25(OH)D deficient pregnant mothers and their newborns and the antenatal visits should include education on the safety and importance of sufficient sunlight exposure during pregnancy. Moreover, pregnancy‐specific 25(OH)D cut‐offs and dietary recommendations are required for optimal health of the mothers and the newborns and to enable comparisons. Well‐designed prospective and interventional studies are required to investigate the optimum Vitamin D requirement for pregnant mothers and their newborns, to thoroughly determine maternal 25(OH)D associations with newborn outcomes and obtain more conclusive results.

## CONFLICT OF INTEREST

The authors declare that they have no conflicts of interest.

## CONTRIBUTIONS

SR and ZH conceived and designed the study. SR, MK, YY and SHH were involved in data collection. SA and SF analysed the data. SA, NL and ZH interpreted the data, revised the manuscript and approved the final draft.
